# Chronic comorbidities and risk of 30-day mortality in hip fracture patients– a Danish National patient registry study

**DOI:** 10.1007/s00068-025-02934-3

**Published:** 2025-08-11

**Authors:** Regitze H. Liebermann, Jes B. Lauritzen, Henrik Løvendahl Jørgensen

**Affiliations:** 1 Department of Orthopedic Surgery, Bispebjerg and Frederiksberg Hospital, Copenhagen, Denmark; 2 Department of Clinical Medicine, University of Copenhagen, Copenhagen, Denmark; 3 Department of Clinical Biochemistry, Hvidovre Hospital, Kettegård Alle 30, Hvidovre, 2650 Denmark

**Keywords:** Hip fracture, Chronic comorbidities, Mortality, Fracture type, Sex

## Abstract

**Purpose:**

Hip fractures are a major health issue among the elderly population and are associated with increased mortality. Chronic comorbidities may cause increased mortality risk among these patients. In this study we examined the impact of chronic comorbidities on 30-day mortality among Danish hip fracture patients along with other risk factors such as sex and fracture type.

**Patients and methods:**

This study used data from the Danish National Patient Registry. We included patients aged ≥ 70 years who sustained a hip fracture between January 1, 1999, and December 31, 2012. Chronic comorbidities– dementia, heart failure, chronic obstructive pulmonary disease, asthma, ischemic heart disease, diabetes mellitus with complications, chronic kidney disease, and cancer– as well as fracture type were identified using ICD-10 codes.

**Results:**

A total of 98,850 hip fracture patients were included, of whom 11.5% had died within 30 days. Male patients had a significantly higher 30-day mortality rate (17.7%) than female patients (9.2%). Patients with multiple chronic comorbidities had an increased mortality risk, with patients having ≥ 5 chronic comorbidities exhibiting a 30-day mortality rate of 28.3% compared to 7.8% for patients without any chronic comorbidities. Regarding fracture types, patients with subtrochanteric fractures had a mortality risk of 13.2% compared to patients with a femoral head/neck fracture, who had a mortality risk of 10.8%. In adjusted analyses chronic kidney disease and male sex had the highest association with increased 30-day mortality risk.

**Conclusion:**

This study shows the significant impact of chronic comorbidities as well as sex and fracture type on 30-day mortality in hip fracture patients. Patients with multiple chronic comorbidities had the highest risk of increased 30-day mortality. These findings underline the importance of interventions such as orthogeriatric care to lower 30-day mortality risk for these patients.

## Introduction

Hip fractures are a prevalent health issue among the elderly part of the population [1] and are associated with an increased risk of mortality [2]. Patients who sustain a hip fracture are often more vulnerable and usually suffer from one or more chronic comorbidities [3]. Therefore, understanding the various risk factors related to increased mortality after sustaining a hip fracture is essential. Numerous comorbidities are linked to an elevated risk of mortality for patients undergoing operative treatment for hip fractures [4], a risk that can be observed both within 30 days and 1 year post-injury [1].

A Danish study, using the death certificate registry, identified several of the most common causes of death among patients who have sustained a hip fracture. In addition to complications directly related to the fracture, these included cardiovascular disease (CVD), pulmonary disease (unspecified), cancer, chronic kidney disease (CKD), diabetes and dementia [1]. The presence of these comorbidities has also been shown in various other studies to influence the risk of mortality.

Another Danish study thus demonstrated that patients with preexisting cardiovascular disease (CVD) who sustained a hip fracture had an elevated risk of 30-day mortality. Among CVD-related comorbidities, ischemic heart disease was identified as the most prevalent, while heart failure exhibited the strongest association with increased mortality within 30 days [5]. A South Korean study comparing hip fracture patients with and without chronic obstructive pulmonary disease (COPD) found that COPD was an independent risk factor for increased mortality at 1-year follow-up [6]. A Swedish study found that, at 3-years follow-up, 13% of deaths among patients with prior hip fractures were attributable to cancer making it one of the most common causes of death after hospital discharge. Additionally, a cancer diagnosis at baseline was identified as a predictor of increased mortality risk [7].

For CKD, a study showed that it was associated with a higher mortality within 2 year after sustaining a hip fracture [8]. A Danish population-based cohort study of hip fracture patients demonstrated increased mortality among individuals with diabetes. Although the increased mortality risk associated with diabetes decreased with advancing age, patients with diabetes continued to exhibit higher mortality rates compared to those without diabetes [9]. A Japanese study examining mortality risk among hip fracture patents with dementia found a significantly higher mortality rate in this group. Additionally, patients with dementia were at increased risk of developing complications such as urinary tract infections (UTIs) and respiratory issues, including pneumonia. These complications, commonly observed after hip fracture surgery, are also associated with elevated mortality risk [10].

The findings from the studies mentioned above indicate that comorbidities influence mortality and can be a part of the prediction of outcomes for hip fracture patients. Consequently, the aim of this study is to examine the impact of chronic comorbidities on mortality among Danish hip fracture patients. Specifically, the study seeks to assess the extent to which chronic comorbidities contribute to 30-day mortality rates within the Danish population.

## Patients and methods

Data was collected from the Danish National Patient Registry (DNPR) through remote access to Statistics Denmark on all patients in Denmark above 70 years, sustaining a hip fracture (fracture of the femoral neck (DS720), pertrochanteric fracture (DS721) or subtrochanteric fracture (DS722)) during the period January 1, 1999, to December 31, 2012. Data on mortality was retrieved 31 December 2013. For patients sustaining more than 1 hip fracture during the period, only the first was included and used as index fracture for survival analysis. A total of 98.850 patients sustained a hip fractures during this period.

Data on comorbidities and fracture types were extracted from the Danish National Patient Registry using the civil registration number of each patient. This 10-digit registration number, assigned to each Danish citizen at birth through the Civil Registration System, enables tracking of individuals across their lifetime and facilitates the retrieval of data from multiple registries. The data was anonymized at Statistics Denmark before analysis.

Based on the literature referenced in the introduction section, we included the most common comorbidities with possible impact on mortality of hip fracture patients in the study: dementia, heart failure (HF), chronic obstructive lung disease (COPD), asthma, ischemic heart disease (IHD), diabetes mellitus with complications (DM), chronic kidney disease (CKD), and cancer. All chronic comorbidities and fracture types were identified based on their corresponding ICD-10 diagnostic codes assigned to the patient at the time of their hospital admission.

### Statistics

The age of the participants was normally distributed and is thus shown as Mean (SD). Differences between the two groups were tested using unpaired t-test. Categorical values are shown as N (%) and differences were tested using chi-square tests.

For each of the included variables, we calculated unadjusted and adjusted odds ratios (ORs) for 30-day mortality with 95% confidence intervals.

Subsequently, a stepwise logistic regression analysis with backwards elimination was performed to develop a model for predicting 30-day mortality. All the non-combined candidate variables (dementia, HF, COPD, asthma, IHD, DM, CKD, cancer, age, sex and type of fracture) from the study were entered into the first step of the analysis. The median chi-square value in the first step was 234.6. A limit of inclusion into the model was set at the 10% percentile corresponding to a chi-square value of 13.2. In the first step, HF (chi-square = 0.6) was excluded. In the second step, asthma (chi-square = 12.8) was excluded. Thus dementia, COPD, IHD, DM, CKD, cancer, age, sex and type of fracture were included in the final model.

For all statistical analyses, a *P*-value of < 0.05 was considered statistically significant.

All statistical analyses were conducted using SAS version 9.4 accessed via a secure remote connection provided by Statistics Denmark.

### Ethics and approvals

According to Danish law, ethical committee approval is not required for this type of register study. The data was obtained through secure remote access to Statistics Denmark (ref. 704670) and the study was approved by the Danish Data Protection Agency (2012-58-0004, local number BBH-2014-050).

## Results

A total of 98,850 patients aged ≥ 70 years were admitted to a hospital in Denmark with hip fractures in the period 1999 to 2012. Among these, 11,333 patients (11.5%) had died within 30 days of admission.

Male patients exhibited a higher 30-day mortality rate of 17.7% compared to 9.2% among female patients (Table 1). The type of hip fracture was also associated with variations in 30-day mortality, with subtrochanteric fractures having the highest risk at 13.2%, compared to femoral head/neck fractures, which had a 30-day mortality risk of 10.8% (Table 1). The presence of chronic comorbidities also influenced the 30-day mortality risk. For each chronic comorbidity in this study, there is an increased percentage of patients dead at 30 days, all results being statistically significant with a *P*-value < 0.0001 except for asthma which had a *P*-value of 0.8. Having multiple chronic comorbidities is also associated with increased mortality as patients with ≥ 5 chronic comorbidities had a 30-day mortality rate of 28.3%, compared to 7.8% for patients without any chronic comorbidities (Table 1).

**Table 1:** Baseline characteristics

	Alive at 30 days	Dead at 30 days	*P*-value
*N*	87517	11333	-
Age	82.8 (6.6)	85.8 (6.7)	*<0.0001*
Male	21788 (82.3)	4699 (17.7)	*<0.0001*
Female	65729 (90.8)	6634 (9.2)	
Fracture type:			
Subtrochanteric	7071 (86.8)	1072 (13.2)	
Pertrochanteric	34542 (88)	4719 (12)	*<0.0001*
Femoral head/neck	45904 (89.2)	5542 (10.8)	
Dementia yes/no	12851 (15)/74666 (85)	2466 (21.8)/8867 (78.2)	*<0.0001*
HF yes/no	1091 (1.3)/86,426 (98.7)	183 (1.6)/11,150 (98.4)	*0.001*
COPD yes/no	8491 (9.7)/79,026 (90.3)	1798 (5.9)/9535 (84.1)	*<0.0001*
Asthma yes/no	2262 (2.6)/85,255 (97.4)	298 (2.6)/11,035 (97.4)	*0.8*
IHD yes/no	16252 (18.6)/71,265 (81.4)	3350 (29.6)/7983 (70.4)	*<0.0001*
DM yes/no	8010 (9.2)/79507 (90.8)	1209 (10.7)/10,124 (89.3)	*<0.0001*
CKD yes/no	1909 (2.2)/85608 (97.8)	667 (5.9)/10,666 (94.1)	*<0.0001*
Cancer yes/no	13899 (15.9)/73618 (84.1)	2415 (21.3)/8918 (78.7)	*<0.0001*
No. of comorbidities:			
0	42702 (92.3)	3587 (7.8)	
1	29681 (87.1)	4391 (12.9)	
2	11228 (82.9)	2322 (17.1)	*<0.0001*
3	3109 (79.0)	827 (21.0)	
4	693 (80.8)	165 (19.2)	
≥5	104 (71.7)	41 (28.3)	

Age: Mean (SD). Categorical variables: *N* (%)

*HF* heart failure, *COPD* chronic obstructive pulmonary disorder, *DM* Diabetes mellitus with complications, *IHD* ischemic heart disease, *CKD* chronic kidney disease

Table 2 shows the number of co-occurrences between the comorbidities. The most frequently co-occurring (more than 2000 patients) comorbidities were: IHD + cancer (*n* = 3470), IHD + COPD (*n* = 3440), IHD + dementia (3216), IHD + DM (*n* = 3084), dementia + cancer (*n* = 2303) and COPD + cancer (*n* = 2093).

**Table 2:** Number of co-occurrences between the comorbidities

	Dementia	HF	COPD	Asthma	IHD	DM	CKD	Cancer
Dementia								
HF	151							
COPD	1405	238						
Asthma	347	58	1642					
IHD	3216	550	3440	823				
DM	1551	244	1176	302	3084			
CKD	385	153	491	89	1141	620		
Cancer	2303	193	2093	496	3470	1761	605	

*HF* heart failure, *COPD* chronic obstructive pulmonary disorder, *DM* Diabetes mellitus with complications, *IHD* ischemic heart disease, *CKD* chronic kidney disease

Table 3 summarizes the calculated odds ratios (OR) for the individual chronic comorbidities as well as for the most frequently co-occurring comorbidities, with both unadjusted and adjusted ORs presented with their 95% confidence intervals (95% CI). In the unadjusted analysis, asthma was not significantly associated with an increased 30-day mortality (OR: 1.02; 95% CI: 0.90–1.15; *P* = 0.8). The unadjusted ORs for all other chronic comorbidities and for the most frequently co-occurring comorbidities were statistically significant (Table 3). In the analysis adjusted for age, sex and fracture type, all the parameters were statistically significant with ORs ranging from 1.22; 95% CI: 1.08–1.39, *P* = 0.002 for asthma to 2.48; 95% CI: 2.26; 2.73, *P* < 0.0001 for CKD (Table 3).

**Table 3:** Unadjusted and adjusted odds ratios for 30 days mortality for each chronic comorbidity, for the most frequently co-occurring (more than 2000 patients) comorbidities and for the number of comorbidities. Adjustment was made for age, sex and fracture type

	Unadjusted OR	*P*-value	Adjusted OR	*P*-value
Dementia	1.62 [1.54;1.70]	*P*<0.0001	1.47 [1.40;1.55]	*P*<0.0001
HF	1.30 [1.11;1.52]	*P*=0.0011	1.33 [1.13;1.57]	*P*=0.0005
COPD	1.75 [1.66;1.85]	*P*<0.0001	2.01 [1.89;2.13]	*P*<0.0001
Asthma	1.02 [0.90;1.15]	*P*=0.8	1.22 [1.08;1.39]	*P*=0.002
IHD	1.84 [1.76;1.92]	*P*<0.0001	1.71 [1.63;1.78]	*P*<0.0001
DM	1.19 [1.11;1.26]	*P*<0.0001	1.31 [1.23;1.40]	*P*<0.0001
CKD	2.80 [2.56;3.10]	*P*<0.0001	2.48 [2.26;2.73]	*P*<0.0001
Cancer	1.43 [1.37;1.51]	*P*<0.0001	1.42 [1.35;1.49]	*P*<0.0001
IHD+cancer	1.91 [1.75;2.08]	*P*<0.0001	1.73 [1.58;1.89]	*P*<0.0001
IHD+COPD	2.09 [1.92;2.27]	*P*<0.0001	2.21 [2.02;2.41]	*P*<0.0001
IHD+dementia	2.22 [2.04;2.42]	*P*<0.0001	1.95 [1.79;2.13]	*P*<0.0001
IHD+DM	1.63 [1.48;1.80]	*P*<0.0001	1.74 [1.57;1.92]	*P*<0.0001
Dementia+cancer	1.72 [1.55;1.92]	*P*<0.0001	1.56 [1.39;1.74]	*P*<0.0001
COPD+cancer	2.01 [1.80;2.23]	*P*<0.0001	2.20 [1.96;2.46]	*P*<0.0001
Number of comorbidities	1.46 [1.43;1.49]	*P*<0.0001	1.46 [1.44;1.49]	*P*<0.0001

*HF* heart failure, *COPD* chronic obstructive pulmonary disorder, *DM* Diabetes mellitus with complications, *IHD* ischemic heart disease, *CKD* chronic kidney disease

**Fig. 1:**
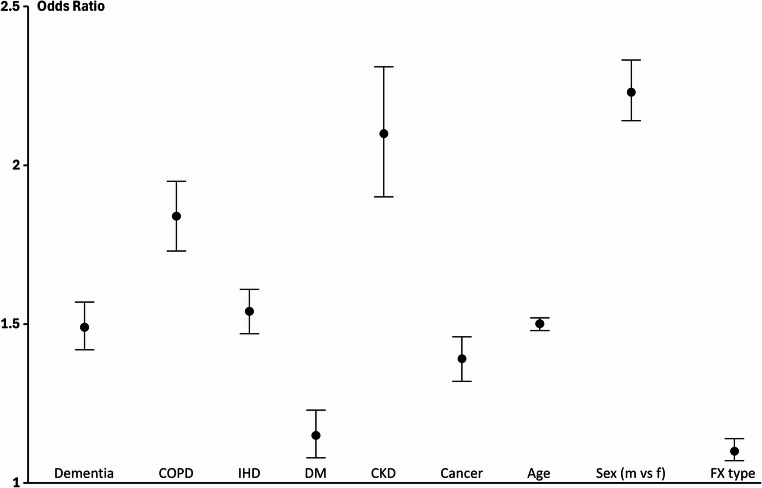
Odds ratios (OR) with confidence intervals (CI) for the various risk factors in the final model after stepwise logistic regression analysis. HF: heart failure, COPD: chronic obstructive pulmonary disorder, DM: Diabetes mellitus with complications, IHD: ischemic heart disease, CKD: chronic kidney disease

Finally, a stepwise logistic regression analysis was performed to determine the ORs with confidence intervals (CI) for the covariates remaining significant in the final step of the analysis: dementia, COPD, IHD, DM, CKD, cancer, age, sex and fracture type. This is displayed in Fig. 1. All risk factors had an OR higher than 1 with CKD and male sex having the highest ORs.

## Discussion

In this study, we wished to investigate the 30-day mortality risk in patients who sustained a hip fracture, focusing on risk factors such as sex, fracture type, and the presence of various chronic comorbidities, including heart failure, chronic obstructive pulmonary disease (COPD), ischemic heart disease (IHD), and chronic kidney disease (CKD).

We found a significant influence of chronic comorbidities on 30-day mortality. Patients with ≥ 5 chronic comorbidities had a significantly higher mortality rate compared to patients with no chronic comorbidities. This aligns with research indicating that higher comorbidity burdens are associated with poorer outcomes following hip fractures. A study assessing the Nottingham Hip Fracture Score (NHFS) thus found that a higher comorbidity burden, measured using the Charlson Comorbidity Index (CCI), was linked to increased mortality at both 30 days and 1 year following a hip fracture [11]. This highlights the importance of relevant geriatric care in patients with multiple chronic comorbidities, as these factors contributes to poorer outcomes after sustaining a hip fracture.

Dementia was found to be a significant risk factor for 30-day mortality in the unadjusted model but lost its significance in the adjusted model. This suggests that the effect of dementia on mortality may be mediated by other factors, such as the presence of other comorbidities or the overall functional status of the patient. A meta-analysis demonstrated that dementia increased 30-day mortality by 1.57 fold in patients undergoing hip-fracture surgery [12]. However, after adjusting for factors like comorbidities and functional status, the direct impact of dementia on mortality may decrease, indicating that its effect could be mediated through other variables.

Our study also identified CKD as a significant risk factor for 30-day mortality following hip fractures. Patients with CKD have been shown to have an increased risk of hip fracture-related mortality. A study by Robertson et al. found that hip fracture incidence was higher in individuals with CKD stages 3–5 compared to those with normal kidney function [13]. Additionally, post-hip fracture mortality was increased in CKD stage 4 [13]. This underscores the need for careful management of hip fracture patients with CKD to improve outcomes.

Our findings further confirm that male patients have significantly higher mortality rate, subtrochanteric fractures are associated with increased mortality, and multiple comorbidities significantly elevates the risk of mortality in hip fracture patients.

This difference in mortality has been reported in other studies as well. For instance, a study from the Swedish Fracture Register found that male sex was associated with both increased 30-day and 1-year mortality following a hip fracture [14]. Similarly, a Danish study also identified male sex as significant risk factor for increased mortality following hip fractures [15]. These findings suggest that male patients may have a higher risk profile, possibly due to factors such as a greater burden of comorbidities.

Finally, we found that subtrochanteric fractures were associated with a higher 30-day mortality rate compared to femoral neck and head fractures. This result is consistent with findings from the Swedish Fracture Register, where they suggested that the higher mortality in patients with subtrochanteric fractures could be related to the more complex surgical treatment of these fracture [14]. This highlights the importance of considering fracture type when evaluating the prognosis, and treatment, and rehabilitation planning for hip fracture patients.

The main strength of this study is the large number of patients included from the Danish National Patient Registry using the Danish civil registration number. This makes it possible to have a complete follow-up regarding mortality and it provides information on the different variables collected unbiasedly with a high degree of validity from the national registries [16].

Since this study is based on data collected from national registries it was not possible to get information on other important confounders that could impact mortality such as treatment during hospitalization, type of surgery, time to surgery, smoking, alcohol consumption, nutritional status, body mass index, functional indices or fragility indices. In our study, there is therefore likely to be residual confounding and further studies including some or all of these variables could further refine prediction algorithms. The study covers a relatively long period from 1999 to 2012. We have previously published a study on the same population which showed that the 30-day mortality remained constant during the study period [3], indicating that new treatments with significant impact on mortality was unlikely to have been introduced during the study period. We did therefore not include secular trends in the statistical models.

Overall, our study underscores the importance of considering sex, fracture type, and chronic comorbidities when assessing mortality risk in hip fracture patients. The higher mortality rates in patients with multiple comorbidities, especially CKD, highlight the need for individualized treatment strategies. Comprehensive geriatric care, which involves multidisciplinary management of medical, surgical and rehabilitative needs, is crucial for improving outcomes in these high-risk populations [17]. Further research is needed to explore the complex interactions between chronic diseases, fracture type, and age to refine more optimal treatment strategies for hip fracture patients. This could include a greater focus on palliative care on patients not expected to survive.

## Conclusion

This study shows the significant impact of chronic comorbidities as well as sex and fracture type on 30-day mortality in hip fracture patients. Patients with multiple chronic comorbidities had the highest risk of increased 30-day mortality. These findings underline the importance of interventions such as orthogeriatric care to lower 30-day mortality risk for these patients.

## Data Availability

No datasets were generated or analysed during the current study.
